# The Trained Nurse

**Published:** 1904-12

**Authors:** 


### THE TRAINED NURSE.

When I was sick I had a trained nurse. She came in the still watches of one evening, and laid her soft, cool, twenty-five-dollar-a-week hand on my burning pauper brow, and thenceforth her salary and my fever ran on together, not even stopping for meals—that is to say, the nurse herself stopped for meals, but not her salary. About noon each day, when the glad outside world was caroling to the sky, when the merry school-boy was skipping homeward, and the flowers were dancing in the sunlight, she would part from me with tears in her eyes and a choking sensation in her throat and a look of keen agony, and slip gently down stairs and spend a few hours over the family board, while the cook threatened to leave and the hot-water bottle on my jaded stomach became frappé.

She came to me with a complete set of books, a clinical thermometer, and the story of her past life. When she had taken away my temperature and gone off with it to some far corner of the room and examined it critically by the light of a tallow-dip and set it down in Ledger B, where I couldn't see it, she picked up her trusty pad and began to write a historical novel, of which I

was the unhappy hero. From that moment I felt that about me there was nothing sacred.

The second day after she came, after all the towels had been used up, and all my ingenious children were paving the back yard with remnants of dry toast, and the doctor had told her all about me that she hadn't been able to find out herself, she began to relate to me the story of her past. Two weeks later the crisis in her story and my fever were both passed. We both survived; but at this late day I have an idea that her story is even now the more robust of the two.

The trained nurse is now a necessity in every modern home. As an antidote to medical science, she has no equal. Dressed in rich, but not too gaudy, bed-ticking, and armed with medals she won in the Crimean War for reading *Punch* aloud to the sick soldiers, she stands over one's bedside like a guardian angel, and no germs can pass the line without giving the countersign.—*Tom Masson in Smart Set.*

